# Flying Machines and Flying Men

**Published:** 1918-12-21

**Authors:** 


### The Hospital

The Workers' Newspaper of Administrative Medicine and Institutional Life, Administration, National Insurance and Health.

No. 1698, Vol. LXV. SATURDAY, DECEMBER 21, 1918. PRICE TWOPENCE

### FLYING MACHINES AND FLYING MEN.

A state of war provides conditions and circumstances which act as powerful stimulants to the inventive and adaptive capacities of the combatants and of those whose duty it is to supply the combatants with fighting apparatus. History exhibits numerous examples of this truth, and when the full record of recent experiences is written many modern illustrations will be added to the list. In one direction these experiences break entirely new ground, as never before has war been conducted with the aid of flying machines, and the introduction and development of these will, without question, be carefully studied by naval and military strategists and tacticians. However bright may be the hope that wars will cease from the earth, the maxim which exhorts us to keep our powder dry will, in all probability, still rule national calculations. Of more immediate and general interest is the question of the effect which the experiences of aviation gained during the war will have on the habits and industries of peace. There is a widespread expectation that developments in this direction will be considerable, and already individual events, as well as probabilities arising from the nature of the case, indicate that this expectation will not be disappointed. Compared with many of his animal competitors, man is a slow-moving machine, for having devoted his anterior limbs to prehensile and allied purposes he is left with only his hinder pair as agents in the art of progression. Against this handicap he has struggled throughout the ages, and has devised and perfected many machines to compensate him for the disability. At long last out of his cunning brain and right hand has come the flying machine, and with it he sees himself able to dispute the pace in the air even as by earlier inventions he has compassed swiftness of movement on land and water. He enters now on a new element, and the war has displayed to all and sundry what conquests and conveniences lie open to his enterprise. No one doubts that great developments are at hand, and our engineers and mechanicians are certain to prove equal to the occasion. Kindred triumphs on earth and sea justify the assurance that travel in the air will ere long not only be swift but also safe and sure, and the educational, economic, and social effects of such a development will be far-reaching.

If, however, flying is to be safe, there must be provided not only the machine but also the man. The proposition is true of machines generally, but it has a special emphasis in relation to the flying machine. In no other instance is the relation between man and machine so close a one, and in none is failure of the man so inevitably linked with complete disaster. Hence, even when the engineers and mechanicians have met successfully the mechanical demands created by the conditions which attend aviation, there will remain an obvious risk unless steps are taken to ensure that pilots duly qualified for their special responsibilities are placed in charge. In this respect the experiences of the war have served us well. They have not only given us more complete and more stable flying machines, but they have, in addition, afforded material for the wise selection and training of flying men. In this way, also, the war has meant a rapidity of progress which in other circumstances might well have taken many years to accomplish. Moreover, apart from the war the definition and development of the flying pilot would doubtless, as in many other mechanical arts, have proceeded in an empirical fashion, and would have been regulated by rule of thumb. But so soon as the fighting value of the airship and the aeroplane became evident there was instant pressure on the combatant services to obtain men suitable for the new arm, and in these circumstances to await the rough verdict of experience would have meant loss of time as well as disaster both to men and machines. Some more or less scientific method of selection thus became necessary, and naturally the task was committed to medical examiners. Even for these, at least at the outset, there was the difficulty of a new enterprise, and the only tests available were necessarily general rather than specific. That a candidate was organically sound was indeed something to the good, and this, of course, could be guaranteed. But whether he was by temperament or by the natural capacities and reactions of his nervous and vascular mechanisms likely to prove himself a capable and reliable pilot was a question which could not be answered by the tests of an ordinary medical examination directed, say, to his status relative to a proposal for life insurance. A new problem had arisen, and appropriate methods had to be devised for its solution. To a very large extent this end has been accomplished, and though the final judgment, here, as in many other directions must be supplied by experience and in practice, medical methods are now in existence on the results of which in any individual case a fairly secure judgment can be founded. The candidate can be estimated not merely in reference to the structural integrity of his organs, but also in relation to the degree and quality of his capacity for adjustment to the conditions which exist or may arise in the course of practical aviation.

The gain from this work must be continued now that flying machines propose to carry merchandise and passengers instead of explosive bombs. Though machine-guns and barrages are no longer terrors of the air, the need of the skilful pilot persists, and this necessity involves the need for medical examiners especially equipped for the task of selection. The lessons learned by those who have undertaken the task during the war must be studied by the profession generally, and a paper on the subject by Dr. G. A. Sutherland in a recent issue of the *Lancet* is a valuable contribution to this end. Medicine has ever new opportunities for service to the community, and to guarantee our flying pilots is a duty of this order.

